# A Comparison of Microbial Water Quality and Diversity for Ballast and Tropical Harbor Waters

**DOI:** 10.1371/journal.pone.0143123

**Published:** 2015-11-17

**Authors:** Charmaine Ng, Thai-Hoang Le, Shin Giek Goh, Liang Liang, Yiseul Kim, Joan B. Rose, Karina Gin Yew-Hoong

**Affiliations:** 1 National University of Singapore, Department of Civil and Environmental Engineering, Singapore, Singapore; 2 Michigan State University, Department of Microbiology and Molecular Genetics, East Lansing, Michigan, United States of America; 3 National University of Singapore Environmental Research Institute (NERI), Singapore, Singapore; University of Aveiro, PORTUGAL

## Abstract

Indicator organisms and antibiotic resistance were used as a proxy to measure microbial water quality of ballast tanks of ships, and surface waters in a tropical harbor. The survival of marine bacteria in ballast tanks appeared to diminish over longer water retention time, with a reduction of cell viability observed after a week based on heterotrophic plate counts. Pyrosequencing of 16S rRNA genes showed distinct differences in microbial composition of ballast and harbor waters. The harbor waters had a higher abundance of operational taxonomic units (OTUs) assigned to Cyanobacteria (*Synechococcus spp*.) and α-proteobacteria (SAR11 members), while marine hydrocarbon degraders such as γ-proteobacteria (*Ocenspirillaes spp*., *Thiotrchales spp*.) and Bacteroidetes (*Flavobacteriales spp*.) dominated the ballast water samples. Screening of indicator organisms found *Escherichia coli* (*E*. *coli*), *Enterococcus* and *Pseudomonas aeruginosa* (*P*. *aeruginosa*) in two or more of the ballast and harbor water samples tested. *Vibrio spp*. and *Salmonella* spp. were detected exclusively in harbor water samples. Using quantitative PCR (qPCR), we screened for 13 antibiotic resistant gene (ARG) targets and found higher abundances of *sul1* (4.13–3.44 x 10^2^ copies/mL), *dfrA* (0.77–1.80 x10 copies/mL) and *cfr* (2.00–5.21 copies/mL) genes compared to the other ARG targets selected for this survey. These genes encode for resistance to sulfonamides, trimethoprim and chloramphenicol-florfenicol antibiotics, which are also known to persist in sediments of aquaculture farms and coastal environments. Among the ARGs screened, we found significant correlations (P<0.05) between *ereA*, *ermG*, *cfr* and *tetO* genes to one or more of the indicator organisms detected in this study, which may suggest that these members contribute to the environmental resistome. This study provides a baseline water quality survey, quantitatively assessing indicators of antibiotic resistance, potentially pathogenic organisms and a broad-brush description of difference in microbial composition and diversity between open oceans and tropical coastal environments through the use of next generation sequencing technology.

## Introduction

The city-state of Singapore has one of the busiest ports in the world receiving an average arrival of 140,000 vessels and 2.33 billon gross tonnes of ballast water per year [1]. These commercial ships use ballast water to provide stability and maneuverability, taking up waters when cargo is being unloaded and discharging ballast waters at ports of call [2]. This ballast water exchange can contain a diverse community of organisms, including invasive species (e.g. toxic dinoflagellates, *Vibro spp*.) and potentially impose an environmental and health threat to coastal regions, where ballast waters are released [3–7]. Drake & Lodge (2004) identified regions in Southeast Asia, which may function as hotspots for invasion due to high shipping activity, which in turn facilitates microbial transport [8]. Asexual reproduction and dormancy are strategies, which aid the survival of non-indigenous invasive microbes under environmental stressed conditions. These traits help preserve invasive microbes in the nutrient depleted, light scarce environments of ballast water tanks, and upon discharge to coastal regions where conditions are more optimal, enables successful colonization by rapid population growth [2].

The deballasting of waters from ships acts as a vehicle for the global distribution of pathogens (and possibly antibiotic resistant forms) and waterborne diseases, which may have an adverse impact on humans, marine animals and the aquatic ecosystem as a whole [2]. Thomson et al. (2003) also showed that horizontal gene transfer of antibiotic resistant genes (ARGs) might occur due to the closed system and long water retention time within ballast tanks [9]. Cell death of antibiotic resistant bacteria (ARB) results in the release of intracellular ARGs, which may eventually be transferred into other viable cells within the microbial community imparting resistances in recipient cells. ARGs are increasingly being recognized as an emerging contaminant hence there is an urgency towards early detection in an effort to prevent widespread dissemination of antibiotic resistance [10].

In a study of bacteriological assessment of ballast waters of six ships docked at Singapore harbors, Joachimsthal et al. (2004) used fluorescence in situ hybridization (FISH) coupled with flow cytometer to enumerate total bacteria, and fluorescent tags to differentiate between Enterobacteria, *Vibrio spp*., and *E*. *coli*. Using these techniques, their findings showed that Enterobacteria (range: 0–1.45 x 10^5^ cells/mL) and *Escherichia coli* (*E*. *coli*) (range: 0–7.87 x 10^5^ cells/mL) cells were not always present in all samples, but *Vibrio spp*. (range: 1.45 x 10^4^–1.07 x 10^5^ cells/mL) cells were [11, 12].

In this study, we investigated the microbial content of mid-ocean exchanged (MOE) ballast waters obtained from grab samples from ballast tanks calling at the port of Singapore, and harbor waters surrounding these ships to evaluate differences in microbial diversity between sample types. We expected the ballast waters to represent the microbiological content of the waters at the sites of ballast water uptake, taking into consideration the possibility of the contribution of biofilms, which may develop on the surfaces of ballast water tanks during the voyage. Collectively, we applied pyrosequencing of tagged 16S rRNA gene amplicons as a semi-quantitative method to elucidate differences in microbial diversity, and a culturing and quantitative PCR (qPCR) based approach to survey for the presence of indicator organisms, ARB and associated resistance genes. Among known invasive species, we were interested in the pathogens rather than phytoplankton. Specifically, we focused on monitoring known opportunistic pathogens including *E*. *coli*, *Enterococcus spp*., *Vibrio spp*., and *Pseudomonas aeruginosa* to determine if levels measured complied with the International Maritime Organization (IMO) guidelines.

## Materials and Methods

### Sampling of ballast and harbor waters

Ballast water samples were collected from three vessels with voyages within the Middle East and Asian regions. Each ballast water sample was acquired in the morning, on-board the docked vessel at the Port of Singapore. Seawater next to the hull of the docked ships was collected on the same morning of ballast water sampling for comparative analysis. Ballast waters 1, 2 and 3, (abbreviated as BW1, BW2 and BW3) were taken from the South China Sea, Gulf of Aden and to the west of South Korea in the East China Sea accordingly. The retention time of BW1, 2 and 3 (from the time of collection from the source ports to docking at the Port of Singapore) were 4, 107 and 11 days, respectively. Harbor waters corresponding to BW1, BW2 and BW3 were abbreviated as HW1, HW2 and HW3. Permission for water sampling was granted by the Maritime Port Authority of Singapore and did not involve endangered and protected species.

Environmental parameters including temperature, pH, salinity and turbidity were measured on site using a YSI probe, model 63 (YSI incorporated, Yellow Springs, Ohio, USA). Using a bucket, approximately 60 L of surface water was hauled up through a manhole leading to the ballast tank. Likewise, 60 L of surface water surrounding the docked vessels was sampled. The water samples were concentrated through hollow-fiber ultrafiltration (Hemodialyzer Rexeed^TM^-25S, Asahi Kasei Medical Co., Ltd) to a final volume of 500 mL according to the method of Hill et al. 2005. A volume of 20 mL of concentrated water samples (equivalent to 2.4 L of original water sample), were used for total DNA extraction. Whole water samples were taken for culturing work.

### DNA extraction

The bacterial DNA from the concentrated samples was extracted with the PowerWater DNA Isolation kit (Mo Bio Laboratories, Inc, Carlsbad, CA). DNA yields and purity were measured using a nanodrop spectrophotometer (NanoDrop 2000, Thermo Scientific, Wilmington, USA). The extracted DNA was used for quantification of ARG and *P*. *aeruginosa* and *Salmonella* species.

### Detection of ARG by PCR

A total of 14 primer pairs were used to detect the presence of ARGs, with one primer pair targeting 16S rRNA gene sequences to detect the total bacterial population (Table 1). These primers were also designed to quantify the target genes in real time or quantitative Polymerase Chain Reactions (PCR). Among these, 10 primers were selected from published work, reporting a good detection and quantification of relevant ARGs in qPCR, while 4 primers were designed in this study to target and quantify the specific ARGs, i.e. *ereA*, *ereB*, *sul1*, and *cfr* genes. These gene sequences were downloaded from the GeneBank Database (http://www.ncbi.nlm.nih.gov/) and primers were designed using PRIMER 3 tool [13]. PCR amplicon sizes were specified in the range of 100–300 base pairs (bp) for qPCR analyses.

**Table 1:** Primer sets used in the detection of ARGs and indicator organisms in ballast and harbor water samples.

Name	Sequence of forward and reverse primers (5'-3')	Target gene/species	Amplicon size (bp)	Annealing temperature (°C)	References
*16S EUB-f*	ACTCCTACGGGAGGCAGCAG	16s ribosomal RNA/all bacteria	180	64	[20, 21]
*16S EUB-r*	ATTACCGCGGCTGCTGG				
*ereA-f*	CTTCACATCCGGATTCGCTCGA	erythromycin esterase type I/all bacteria	296	60	In this study
*ereA-r*	ATGGACGCCAACAAGTGAGT				
*ereB-f*	GAATGCAGGGCGATATGGGT	erythromycin esterase type II/all bacteria	171	60	In this study
*ereB-r*	TTTGGCCCATTGGTAGGCAA				
*ermA-f*	CAAGAACAATCAATACAGAGTCTAC	erythromycin resistance methylase/all bacteria	157	60	[22]
*ermA-r*	AGTCAGGCTAAATATAGCTATC				
*ermB-f*	GGTTGCTCTTGCACACTCAAG	erythromycin resistance methylase/all bacteria	191	62	[22]
*ermB-r*	CAGTTGACGATATTCTCGATTG				
*ermC-f*	AATCGTGGAATACGGGTTTGC	erythromycin resistance methylase/all bacteria	293	62	[22]
*ermC-r*	CGTCAATTCCTGCATGTTTTAAGG				
*ermF1-f*	TCGTTTTACGGGTCAGCACTT	erythromycin resistance methylase/all bacteria	182	62	[23]
*ermF1-r*	CAACCAAAGCTGTGTCGTTT				
*ermG-f*	GTGAGGTAACTCGTAATAAGCT	erythromycin resistance methylase/all bacteria	255	61	[22]
*ermG-r*	CCTCTGCCATTAACAGCAATG		255		[22]
*ermT1-f*	GTTCACTAGCACTATTTTTAATGACAGAAGT	erythromycin resistance methylase/all bacteria	124	60	[24]
*ermT1-r*	GAAGGGTGTCTTTTTAATACAATTAACGA				
*sul1-f*	CACCGTTGGCCTTCCTGTAA	sulfonamide resistance gene type I/all bacteria	180	61	In this study
*sul1-r*	TCTAACCCTCGGTCTCTGGC				
*sul2-f*	GCGCTCAAGGCAGATGGCATT	sulfonamide resistance gene type II/all bacteria	293	60	[25]
*sul2-r*	GCGTTTGATACCGGCACCCGT				
*dfrA1-f*	ACGGATCCTGGCTGTTGGTTGGACGC	trimethoprim resistance gene/all bacteria	237	60	[26]
*dfrA1-r*	CGGAATTCACCTTCCGGCTCGATGTC				
*tetM-f*	CC[TA]AC[AT]GTCATTTATATGGA[GA]AGACC	tetracycline resistance gene/all bacteria	304	62.5	[27]
*tetM-r*	CGAAAATCTGCTGG[CGA]GTACT[GA]ACAGGGC				
*tetO-f*	AAGAAAACAGGAGATTCCAAAACG	tetracycline resistance gene/all bacteria	75	60	[28]
*tetO-r*	CGAGTCCCCAGATTGTTTTTAGC				
*cfr-f*	GCTCTAGCCAACCGTCAAGT	Linezolid/chloramphenicol resistance gene/all bacteria	195	61	In this study
*cfr-r*	TCAATTTGCTGCGTTCCTCAC				
*tdh-f* (toxin)	GTAAAGGTCTCTGACTTTTGGAC	*tdh* (toxin)/*V*. *parahaemolyticus*	269	60	[29, 30]
*tdh-r (toxin)*	TGGAATAGAACCTTCATCTTCACC				
*cth-f*	TTCCAACTTCAAACCGAACTATGAC	*cth/V*. *vulnificus*	205	65	[29, 31]
*cth-r*	ATTCCAGTCGATGCGAATACGTTG				
16S-23S rDNA ISR-f	TTAAGCGTTTTCGCTGAGAATG	16S-23S rDNA ISR/*V*. *cholerae*	295	60	[29, 32, 33]
16S-23S rDNA ISR-r	AGTCACTTAACCATACAACCCG				
*regA-f*	TGCTGGTGGCACAGGACAT	*regA/P*. *aeruginosa*	65	60	[34]
*regA-r*	TTGTTGGTGCAGTTCCTCATTG				
*regA-p*	FAM-CAGATGCTTTGCCTCAA-BHQ				
*invA-f*	CGTTTCCTGCGGTACTGTTAATT	*invA/Salmonella spp*.	67	60	[34]
*invA-r*	AGACGGCTGGTACTGATCGATAA				
*invA-p*	FAM-CCACGCTCTTTCGTCT-BHQ				

All PCR assays were performed on a Veriti 96-Well Fast Thermal Cycler (Applied Biosystem, CA, USA). Each PCR reaction included 2 μL 10× Faststart PCR buffer (Roche, IN, USA), 0.25 mM dNTPs (Promega, Singapore), 0.0375 U/μL Taq Faststart, DNA polymerase (Roche, IN, USA), 0.5 mM forward and reverse primers each (AITBiotech, Singapore), 20–200 ng genomic DNA template, with the final volume adjusted to 20 μL using diethylpyrocarbonate (DEPC)-treated distilled deionized water (PureLab Option, Elga, Singapore). The thermo cycles for PCR amplifications were carried out under the following settings: 95°C for 5 min; repeated 40 times with a cycle of denaturation at 95°C for 30 s, at annealing temperatures specific for each primer set for 30 s, elongation at 72°C for 30 s, and finally 72°C for 10 min [14]. ARGs were chemically synthesized (AITBiotech, Singapore) and the gene products served as a positive control. For a negative control, the genomic DNA of a susceptible *E*. *coli* strain (not containing these ARGs) was used. PCR products were visualized using DNA electrophoresis on a 2% agarose gel stained with GelRed^TM^ (Biotium, Inc., Hayward, CA). The gel was visualized under UV light with an E-Gel® Imager System (Life Technologies, Carlsbad, CA).

### Quantification of 16S rRNA genes and ARGs by Real-time qPCR

The calibration curves for qPCR quantification for 16S rRNA genes and each ARG were built using the ten-fold dilution series of ARG standard DNA. Triplicate samples were used for each dilution. Each qPCR reaction contained 10 μL SYBR Select Master Mix (Applied Biosystem, CA, USA), 0.25 μL 10 mM forward primers and reverse primers each, 2 μL DNA template, and 7.6 μL DEPC-treated distilled deionized water. qPCR reactions were performed in a StepOnePlus Real-Time PCR system (Applied Biosystem, CA, USA) with the following settings: holding at 50°C for 2 min and 95°C for 2 min; 40 cycle amplification under conditions of 95°C for 15 s with annealing and elongating at 60°C for 1 min, and finally, a melting curve analysis at 95°C for 15 s, 60°C for 1 min, and 95°C for 15 s. The amplification profiles for each series of DNA dilutions during 40 cycles were recorded. A threshold cycle (Ct) value was determined as the cycle number where fluorescence data crossed the threshold line, which is manually defined within the logarithmic increase phase. The gene copies of 16S rRNA genes and ARGs were calculated as previously described [15]. The molecular weight of a gene was determined by multiplying the size of a gene in base pairs and the average molecular weight of a double-stranded DNA base pair (649 Da). Absolute gene abundance was calculated as gene copies normalized to the volume of ballast and harbor water samples, and relative gene abundance was calculated as percentage of ARGs normalized to the abundance of 16S rRNA gene.

### Determining the phenotypic concentrations of ARB

Marine agar (BD Diagnostics, Maryland, USA) was used to estimate the concentrations of viable and culturable heterotrophic bacteria. Marine agar plates were supplemented with antibiotics to determine the concentrations of ARB. The antibiotics (purchased from Sigma-Aldrich, USA) of the following concentrations were used in the screening process; trimethoprim (4 μg/mL), sulphanilamide (300 μg/mL), norfloxacin (10 μg/mL), lincomycin (100 μg/mL), tetracycline (35 μg/mL), erythromycin (100 μg/mL), kanamycin (80 μg/mL), and linezolid (8 μg/mL). Concentrations used for screening were higher than published minimum inhibitory concentration (MIC) breakpoints in the 2013 Clinical and Laboratory Standards Institute (CLSI) performance standards for Antimicrobial Susceptibility Testing report. Raw water samples were serially diluted in M9 minimal media and 100 μL of samples were spread plated and incubated at 35°C for 24 h and subsequently at room temperature for an additional 24 h. Plates containing colonies within the range of 30–100 cells were counted.

### Measuring indicator organisms using IDEXX kits and qPCR

Colilert^TM^ and Enterolert^TM^ (IDEXX Laboratories, Inc., Westbrook, Maine, USA) were used to quantify the cell numbers of *E*. *coli* and *Enterococcus*, respectively. 100 mL of raw water and a 10 fold diluted raw water sample were tested to allow a wider range of detection due to high fluctuations in bacterial concentration. The water sample was mixed with the Colilert^TM^ or Enterolert^TM^ reagent in a sterile bag and poured into a multi-well tray (Quanti-Tray/2000). The results were expressed as the most probable number (MPN) according to the presence of fluorescence in individual wells when visualized under long-wave ultraviolet light (365 nm) after 24 h of incubation at 37°C (for Colilert^TM^) and 41°C (for Enterolert^TM^).

### Enumeration of pathogenic bacteria

*Vibrio spp*. were enumerated according to the method of Dickinson et al. (2013) with some modifications. Briefly, ballast and harbor water samples at different dilutions (0.1mL, 1mL, 10mL and 100mL) were used to determine the most probable number (MPN) by enrichment in alkaline peptone water (Sigma-Aldrich, St Louis, MO). The samples were incubated overnight at 37°C. A volume of 1 mL of enriched sample was centrifuged at 10 000 g for 10 min to pellet the culture, which was subsequently resuspended in 200 μL of nuclease-free water and heated to boiling for 10 min to release the DNA for the PCR analysis. The primers used for the detection of *Vibrio spp*. are listed in Table 1. PCR was carried out in a thermal cycler (Eppendorf, Germany) and performed in 25 μL volumes consisting 12.5 μL of 2x GoTaq® master mix (Promega, Madison, WI), 1.25 μL of each primer (10 μM), 7.5 μL of nuclease-free water and 2.5 μL of extracted DNA from the *Vibrio spp*. cultures. PCR annealing temperatures and durations for different target *Vibrio spp*. are shown in Table 1. The conditions for PCR amplification was an initial denaturing cycle at 94°C for 2 min, followed by 30 cycles of denaturation at 94°C for 30 s, at an annealing temperature as stated in Table 1 for 1 min and extension at 72°C for 1 min. A final extension step at 72°C for 5 min was also included at the end of 30 cycles. The amplified products were separated by electrophoresis on a 1.5% agarose gel stained with GelRed^TM^ (Biotium, Inc., Hayward, CA). The separated fragments on the agarose gel were visualized under UV light with the E-Gel® Imager System (Life Technologies, Carlsbad, CA).

*P*. *aeruginosa* and *Salmonella spp*. were measured with real-time qPCR method. Primer and probe sets for both *P*. *aeruginosa* and *Salmonella spp*. are summarized in Table 1. A 20 μL qPCR reaction was prepared which contained 10 μL of FastStart Universal Probe Master (ROX) (Roche, Germany), 2 μL of forward and reverse primer each, 0.5 μL of probe, 0.5 μL of nuclease-free reagent-grade water and 5 μL of DNA template. Each set of reactions included both positive (recombinant plasmid DNA) and negative controls (double-deionized water). qPCR amplification for both targets was performed on StepOnePlus^TM^ Real-time PCR system at 50°C for 2 min, 95°C for 10 min, and 45 cycles of 95°C for 15 s and 60°C for 1 min. The threshold cycle values (Ct) obtained from qPCR analysis were converted to gene copies (GC) based on the equations generated from standard curves [*Salmonella spp*.: Ct = -3.4651 log (GC/μl) + 40.416; *P*. *aeruginosa*: Ct = -3.341 log (GC/μl) + 42.466].

### 16S rRNA tag encoded gene sequencing and data analysis

To determine microbial community composition, bacterial tag-encoded FLX amplicon pyrosequencing (bTEFAP) using primers 515F (5’-CACGGTCGKCGGCGCCATT-3’) and 806R (5’-GGACTACHVGGGTWTCTAAT-3’) targeting the V4 region of the 16S rRNA gene was performed using the Roche 454 FLX Titanium platform [16], at the Molecular Research LP (Texas, USA). Datasets obtained from the analyses were processed with using version 1.33.3 MOTHUR [17]. Sequences were trimmed to remove barcodes and primers, quality checked to have an average Phred quality score of 35 or above, with sequences exceeding a homopolymer stretch of more than 8 bases being excluded from the dataset. Sequences were aligned by MOTHUR against the SILVA bacterial reference database (14,956 sequences), screened, filtered and de-noised. UCHIME was used to identify and remove chimeric sequences. Datasets for each sample were normalized by subsampling each dataset to 3,230 reads [18]. Operational taxonomic units (OTUs) were assigned at a distance of 3% using an average neighbor-clustering algorithm. Community diversity between samples was assessed using the observed number of OTUs, Shannon and Simpson indices. Rarefaction curves were generated using MOTHUR and taxonomy of OTUs were assigned using the SILVA database with abundances expressed as percentage of sequences assigned to specific taxa at the phyla, class and family level over the total number of sequenced reads for each sample.

### Statistical analysis

Environmental and biological datasets were analyzed using the statistical software package Primer version 6 [19]. A square-root transformation was applied to the OTU abundance, ARB, ARG, indicator organisms, pathogenic bacteria and environmental datasets. Bray-Curtis similarity values were calculated for the OTU dataset to determine the extent of similarity of microbial community composition between samples and visualized as a dendrogram. A SIMPER analysis was used to determine the taxa, which defined difference in the harbor and ballast water samples. An ANOSIM test was used to verify the significance of observed differences in the microbial community structure. A Principal component analysis (PCA) was used to determine the relationship between the microbial community structure (based on bray-curtis similarity indices), and other biological and environmental parameters. Only parameters that had a Pearson correlation coefficient of >0.7, with a significance of <0.05 were represented in the plot.

## Results

### Bacterial cell and gene counts

**Fig 1:**
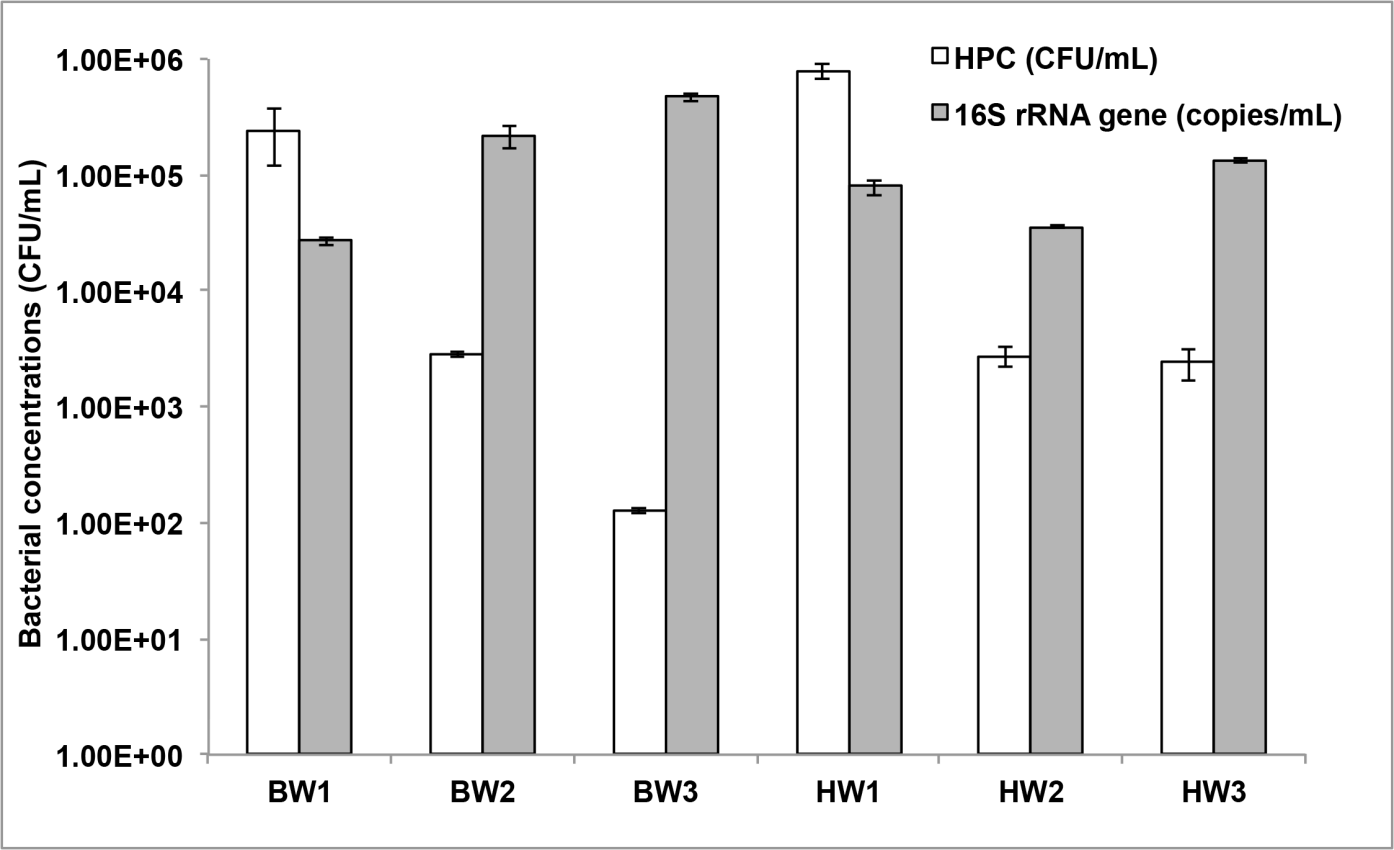
Concentrations of culturable bacteria and total 16S rRNA genes in ballast and harbor waters.

To estimate the concentrations of viable bacterial cells in the two sample types, whole water fractions were plated on marine agar. The average (n = 3) heterotrophic plate counts (HPC) in ballast waters (4.31 x 10^3^ CFU/mL) was approximately a magnitude lower than harbor waters (1.06 x 10^4^ CFU/mL, Fig 1). When determining concentrations by qPCR for total bacterial populations including both the culturable and non-culturable bacteria, the average 16S rRNA gene copies in ballast samples (1.40 x 10^5^ CFU/mL) were higher compared to the harbor waters (7.24 x 10^4^ CFU/mL). Discrepancies in the counts of viable HPC and total bacterial populations within ballast water samples could be due to long retention time and thus, nutrient depletion and reduction of viable cells in the ballast tank over the voyage. This is evident in BW2 and BW3 samples where the ballast waters had a longer retention time (107 and 11 days, respectively), and showed higher 16S rRNA gene copy concentrations and lower HPC compared to BW1 samples, which were held in the ballast water tanks for 4 days. Overall, two of the ballast waters in our study had significantly lower concentrations of viable HPC than harbor waters (with exceptions of BW2 and HW2).

### Microbial diversity of ballast and harbor waters

**Fig 2:**
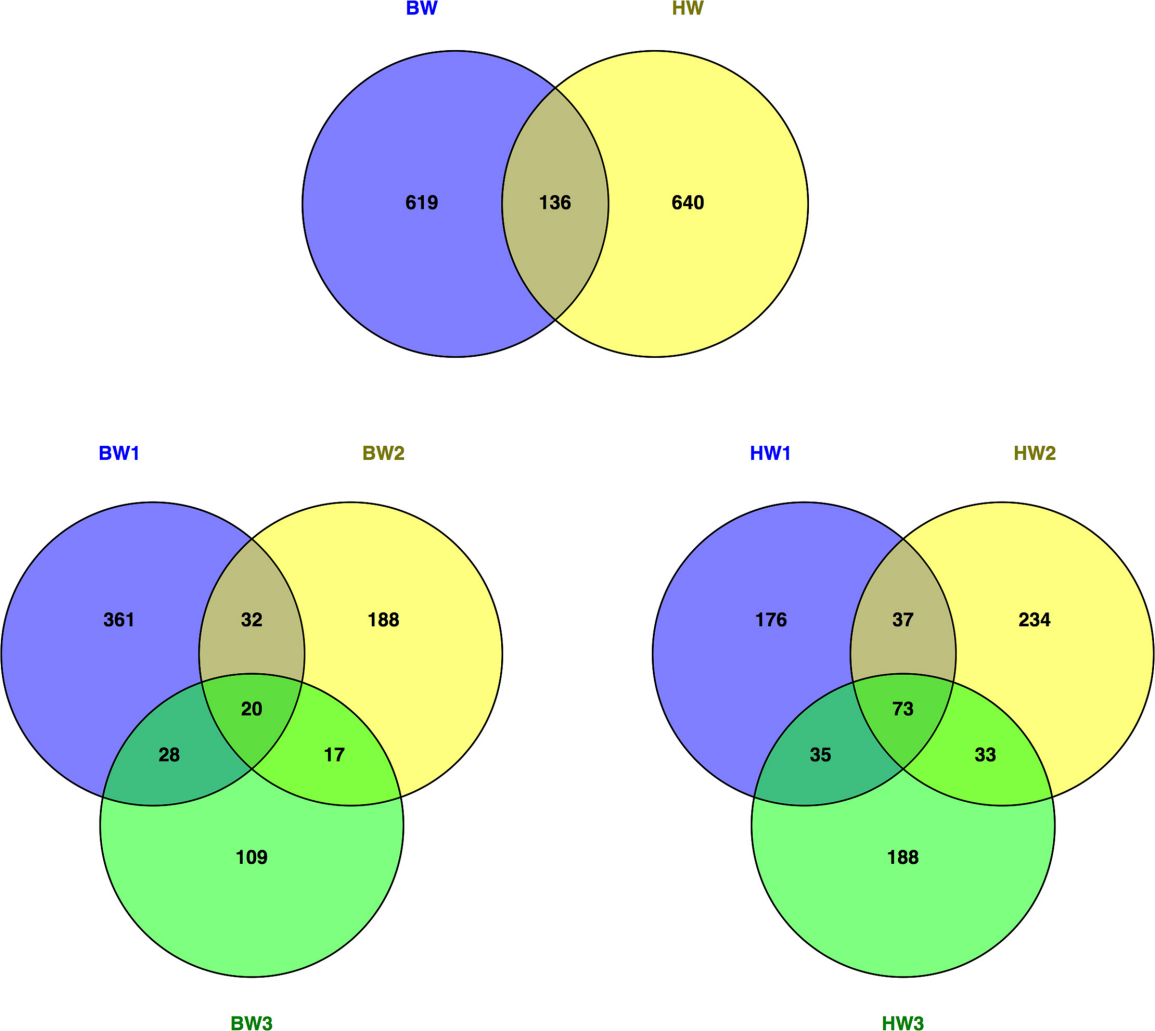
Total OTUs identified in ballast (BW) and harbor waters (HW) from community sequencing analysis.

A total of 57,576 sequences assigned to 1,395 unique OTUs were identified at the genus level (97% similarity cut off) from all samples. Sequences were deposited into the NCBI SRA archive website (http://www.ncbi.nlm.nih.gov/) and available under SRA accession number SRP065174. There were 136 OTUs shared by microbial communities, with 619 and 640 OTUs exclusive to the ballast and harbor communities, respectively (Fig 2). Within the ballast and harbor samples, the harbor waters were more similar in microbial composition (73 common OTUs) in comparison to the ballast waters (20 common OTUs).

Rarefaction curves indicated that a sufficient number of sequences were sampled to cover the majority of species in the microbial communities (S1 Fig). Shannon and Simpson indices showed that BW1 had highest species diversity (Shannon– 4.315, Simpson– 0.9501) and richness (441 OTUs) compared to the two ballast water samples which had values lower than those of the HW samples (Table 2). In general, the HW samples appeared to be more diverse than the BW samples with the exception of BW1, which was taken up at from the South China Sea.

**Table 2:** Diversity of microbial community composition between samples.

Sample	No. of OTUs	No. of sequences	Shannon diversity	Simpson index
BW1	441	3230	4.315	0.9501
BW2	257	3230	2.752	0.7060
BW3	174	3230	2.670	0.8153
HW1	321	3230	3.522	0.9076
HW2	377	3230	3.590	0.9032
HW3	329	3230	3.984	0.9499

**Fig 3:**
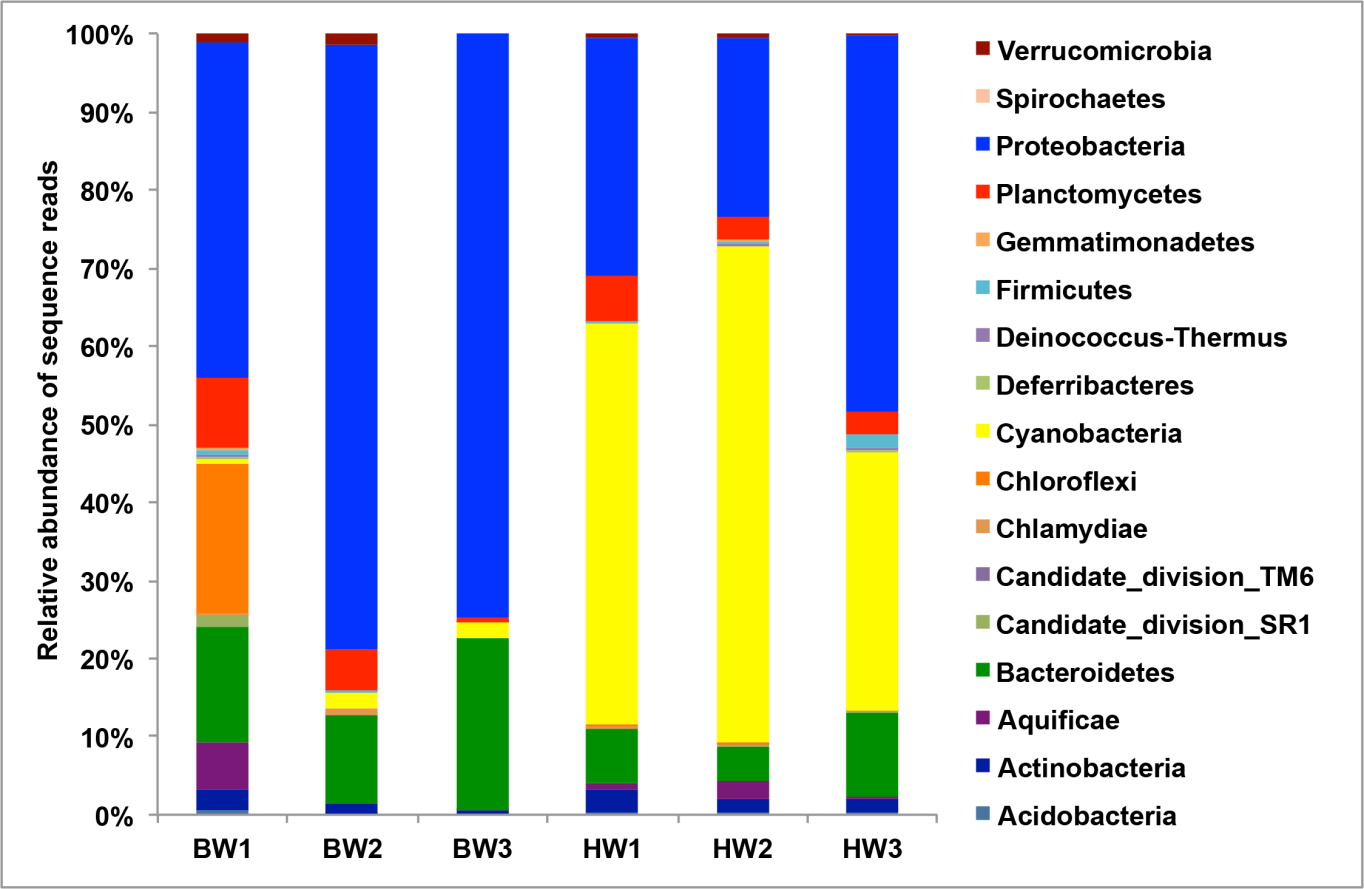
Relative abundance of total sequences assigned at the phyla level.

Dominant taxa representing an average of >1% of total assigned sequences across all sample types included Proteobacteria (49.36 ± 22.54), Cyanobacteria (25.34 ± 27.84), Bacteroidetes (11.78 ± 6.25), Planctomyces (4.43 ± 2.93), Chloroflexi (3.39 ± 7.78), Actinobacteria (1.80 ± 0.88) and Aquificae (1.59 ± 2.32) (Fig 3). A SIMPER analysis showed an average dissimilarity of 52% between the ballast and harbor water sample types. The harbor waters were enriched in Cyanobacteria sequences (~30 fold) while ballast waters had more Proteobacteria (~2 fold), Bacteroidetes (~2 fold) and Chloroflexi (~18 fold) sequence. A SIMPER analysis showed that these taxa accounted for 90% of the total dissimilarity between the microbial communities of the two sample types.

At the family level, among the dominant phyla, Sphingomonadales, Oceanospirillales, Thiotrichales and Flavobacteriales were more represented in the ballast waters (Table 3). The higher relative abundance of Chloroflexi differentiated BW1 among the other water samples, and deeper inspection of the sequence reads showed that Caldilineales was the main contributor to the abundance. One of the striking differences between the ballast and harbor water sample types was the prevalence of SAR11 and *Synechococcus spp*. in the harbor waters.

**Table 3:** Percentage of total sequences assigned to most dominant taxa. Data presented include the top 4 abundant phyla/classes across samples.

Phyla/Class	Family/Genera	BW1	BW2	BW3	HW1	HW2	HW3
α-proteobacteria	Sphingomonadales	7.99	5.20	1.42	0.06	1.27	3.41
	SAR11	1.89	0.00	2.41	16.78	8.14	6.01
	Rhodobacterales	1.39	3.37	1.86	0.50	1.21	4.80
γ-proteobacteria	Alteromonadales	5.14	2.57	3.96	2.51	4.86	16.28
	Oceanospirillales	13.10	1.67	25.11	2.11	1.46	3.59
	Pseudomonadales	0.28	0.06	0.09	2.35	0.40	2.85
	Thiotrichales	1.49	54.46	36.16	0.09	0.09	0.09
Bacteroidetes	Flavobacteriales	3.62	10.68	21.20	6.16	3.38	10.40
	Sphingobacteriales	11.18	0.71	0.93	0.62	1.08	0.40
Chloroflexi	Caldilineales	18.95	0.00	0.00	0.25	0.25	0.03
Cyanobacteria	Synechococcus	0.03	0.06	1.18	42.94	46.01	29.07
	Unclassified	0.37	1.83	0.06	7.89	10.65	3.59

Family/genera representing >1% of total sequences assigned were selected for comparison.

### Quantification of indicator organisms

*Enterococcus* and *E*.*coli* counts were higher in harbor water samples, with the exception of BW3. Viable indicator organisms were detected in the harbor water samples and together with BW3 (from the Gulf of Aden) exceeded the acceptable levels of (100 CFU/100 mL) of intestinal *Enterococci* according to IMO guidelines. *E*. *coli* was detected in BW1 but at levels within the acceptable range. Indicator organisms were present in all the harbor water samples at levels higher than those in the ballast samples. *Vibrio* species (including toxin-producing species) were below the detection limit in all ballast water samples, but were detected in two of the harbor water samples (HW1 and HW2, Table 4). *V*. *parahaemolyticus* was absent in all samples, however *V*. *cholerae* and *V*. *vulnificus* were detected in two of the harbor water samples. MPN-PCR results showed that BW1 and BW2 were positive for *P*. *aerunginosa* with a relatively low concentration of 19–20 MPN/100 mL in comparison to two harbor samples, HW1 and HW2, which had much higher concentrations (1,322–19,393 MPN/100 mL, Table 4). Higher levels of *P*. *aeruginosa* gene copies were detected in two of the harbor water samples (HW1 and HW2) with lower levels (100–1,000 fold less) in the ballast waters (BW1 and BW2, Table 4).

**Table 4:** Indicator organism measurements in ballast and harbor waters using IDEXX kits and qPCR methods.

Method of detection	Target	BW1	BW2	BW3	HW1	HW2	HW3
IDEXX kit (MPN/100mL)	*E*. *coli*	140	6	0	2,603	893	471
IDEXX kit (MPN/100mL)	*Enterococcus*	45	51	251	1,908	222	135
PCR (MPN/100mL)	*V*. *cholera*	0	0	0	110	24	0
PCR (MPN/100mL)	*V*. *parahaemolyticus*	0	0	0	0	0	0
PCR(MPN/100mL)	*V*. *vulnificus*	0	0	0	110	110	0
qPCR(GC/100mL)	*P*. *aeruginosa*	19	20	0	1,322	19,393	19
qPCR (GC/100mL)	*Salmonella spp*.	0	0	0	17	0	0

### Detection of ARBs and ARGs

**Fig 4:**
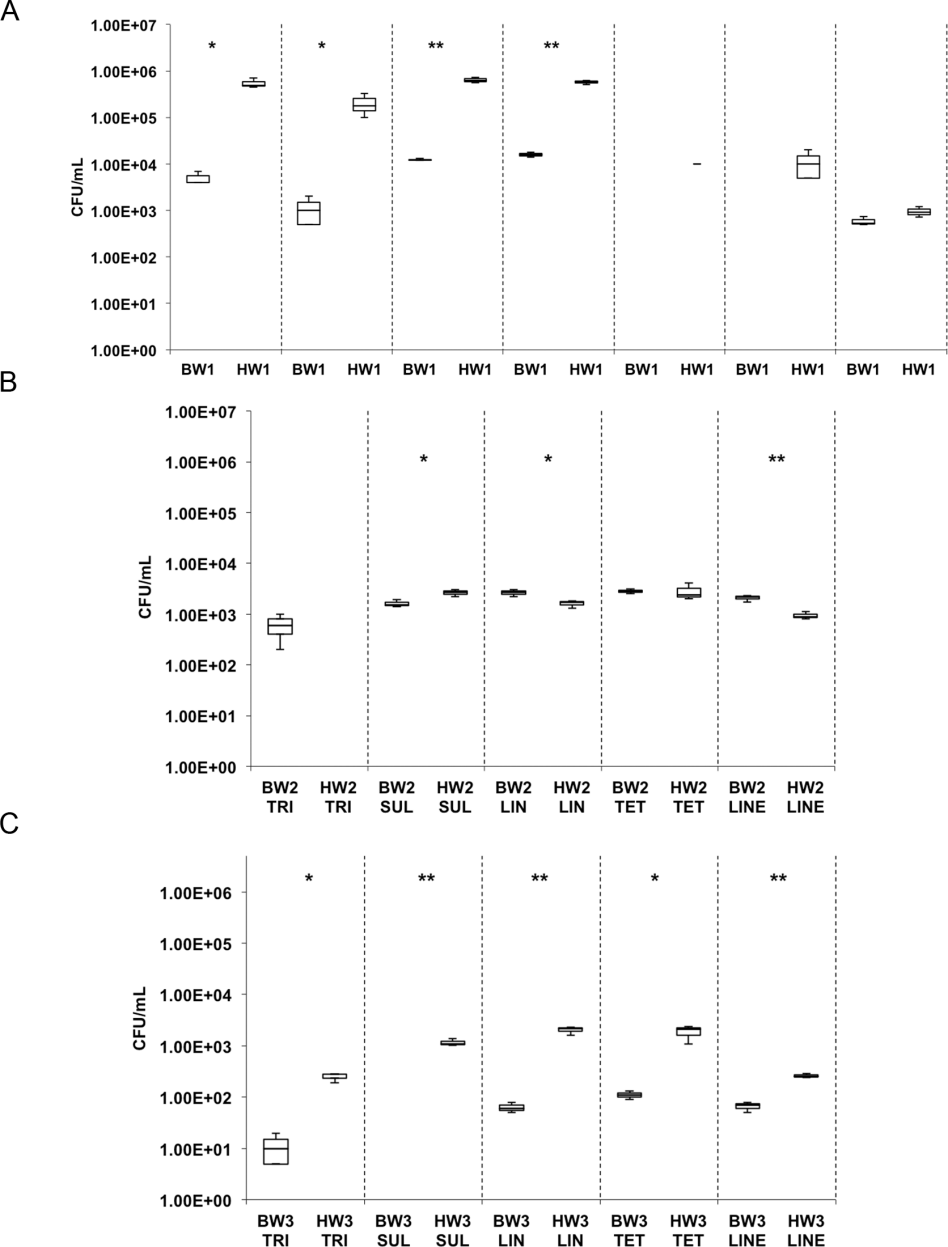
The concentrations of heterotrophic bacteria and their resistance to 8 antibiotics in ballast and harbor waters (A) BW1 and HW1, (B) BW2 and HW2, (C) BW3 and HW3. TRI: Trimethoprim; SUL: Sulfanilamide; NOR: Norfloxacin; LIN: Lincomycin; TET: Tetracycline; ERY: Erythromycin; KAN: Kanamycin; LINE: Linezolid. Two-tailed T-test, *P<0.05, **P<0.01.

There was no colony growth on norfloxacin-supplemented plates across all samples. BW1 and HW1 samples showed resistance to the remaining 7 antibiotics while BW2 and BW3 as well as HW2 and HW3 showed resistance to 5 antibiotics (Fig 4). In most cases, the ballast water samples had significantly lower counts of ARB compared to the harbor water samples (Fig 4). Among the harbor water samples, HW1 had the highest resistant counts to trimethoprim, sulfanilamide and lincomycin (1.8–7.0 x 10^5^ CFU/mL).

**Fig 5:**
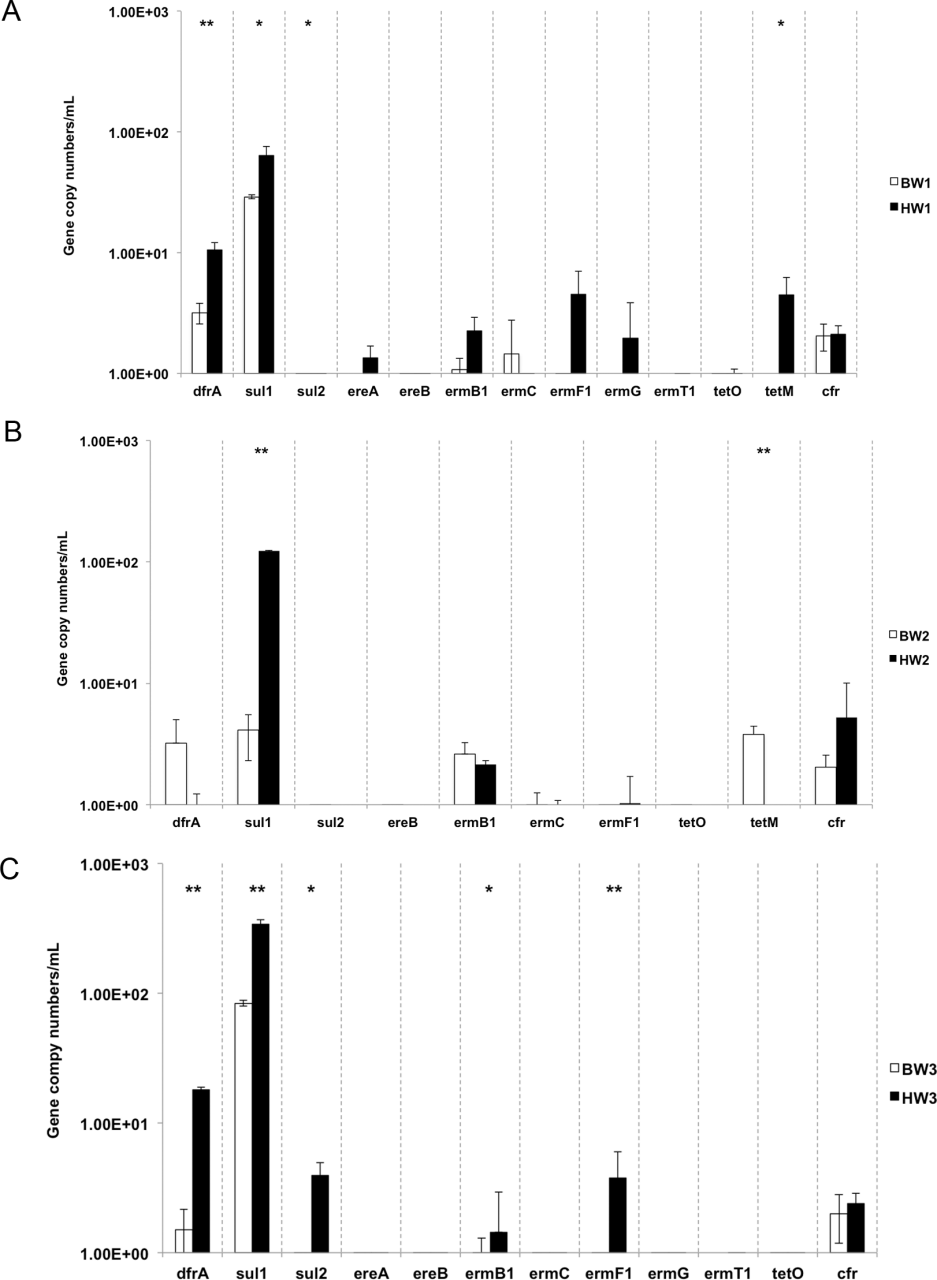
The abundance of the ARGs in ballast and harbor waters. (A) BW and HW1, (B) BW2 and HW2, (C) BW3 and HW3. Two-tailed T-test, *P<0.05, **P<0.01. ARGs with non-detects in both BW and HW samples were excluded.

Genes linked to resistance of sulfanamide (*sul1*), trimethoprim (*dfrA*) and chloramphenicol-florfenicol (cfr) were most abundant across all samples with levels ranging from 4.13–3.44 x 10^2^, 0.77–1.80 x 10^1^, 2.00–5.21 gene copies/mL respectively (Fig 5). *Sul1* genes were at higher concentrations in harbor waters, while *sul2* and *dfrA* concentrations were higher in the harbor waters with the exception of HW2. In general, harbor water samples had higher measurements of ARGs in comparison to the ballast waters.

### Correlations between microbial community composition, environmental variables, indicator organism loads and antibiotic resistance

The pH of water samples ranged between 5.98–7.87, with HW2 having lowest value measured (pH 5.98, Table 5). HW1 and HW2 samples also showed slightly higher temperatures and turbidity measurements than the rest of the samples. HW1 had the lowest salinity levels, which could be attributed to dilution due to rainfall prior to sampling. A Pearson correlation of environmental variables to ARG, ARB and indicator organisms showed negative correlations between pH and *crf* gene copies, linezolid resistant counts and *P*. *aeruginosa* gene copy numbers (P<0.05, Table 6). Temperature and turbidity measurements were positively correlated to *V*. *vulnificus* (P<0.05, Table 6). Significant correlations were found between the ARGs *eraA*, *ermG*, *cfr* and *tetO* and indicator organisms (P<0.05, Table 7).

**Table 5:** Physical characteristics of ballast and harbor waters.

Sample	Water residence time (days)	Sampling date	Temperature (°C)	pH	Turbidity (NTU)	Salinity(ppt)
BW1	4	14/5/2014	29.9	6.9	0	14
BW2	107	21/5/2014	30.0	7.85	0	20.7
BW3	11	25/5/2014	29.8	7.71	4	18.9
HW1	N/A	14/5/2014	30.3	7.63	10	11.8
HW2	N/A	21/5/2014	30.5	5.98	10	27.6
HW3	N/A	25/5/2014	30.2	7.87	0	30.9

**Table 6:** Pearson correlation analysis of environmental variables to ARG, ARB and indicator organism measurements from ballast and harbor waters.

Variable (units)	Temperature (°C)	pH	Turbidity (NTU)	Salinity (ppt)
*cfr* gene (gene copies/mL)	0.755 (0.082)	**-0.854** * **(0.030)**	0.576 (0.231)	0.530 (0.280)
LINE ARB concentrations (CFU/mL)	0.544 (0.265)	**-0.893** * **(0.017)**	0.467 (0.350)	0.122 (0.818)
*E*. *coli* (MPN/100mL)	0.627 (0.182)	-0.053 (0.920)	0.756 (0.082)	-0.347 (0.501)
*Enterococus spp*. (MPN/100mL)	0.366 (0.475)	0.171 (0.746)	0.666 (0.148)	-0.541 (0.267)
*V*. *cholerae* (MPN/100mL)	0.506 (0.306)	0.009(0.986)	0.746 (0.089)	-0.491 (0.322)
*V*. *vulnificus* (MPN/100mL)	**0.832** * **(0.040)**	-0.536 (0.273)	**0.949** * **(0.004)**	-0.099 (0.853)
*P*. *aeruginosa* (gene copies/mL)	0.743 (0.090)	**-0.875** * **(0.022)**	0.648 (0.165)	0.422 (0.405)
*Salmonella spp*. (gene copies/mL)	0.340 (0.509)	0.201 (0.703)	0.600 (0.208)	-0.580 (0.227)

P-values are represented in parenthesis with

*P<0.05.

**Table 7:** Pearson correlation analysis of ARGs to indicator organisms from ballast and harbor waters.

Variable	*ereA*	*ereB*	*ermB1*	*ermC*	*ermF1*	*ermG*	*sul1*	*sul2*	*dfrA*	*cfr*	*tetO*
*E*. *coli* (cor. coefficient)	0.998*	-0.181	0.415	-0.285	0.785	0.993	-0.002	-0.037	0.342	0.113	0.957*
*E*. *coli* (p value)	**(0.037)**	(0.732)	(0.413)	(0.584)	(0.064)	(0.075)	(0.997)	(0.945)	(0.507)	(0.831)	**(0.003)**
*Enterococcus* (cor. coefficient)	0.959	-0.110	0.331	-0.412	0.712	0.994	-0.142	-0.116	0.293	-0.149	0.981*
*Enterococcus* (p value)	(0.182)	(0.835)	(0.522)	(0.416)	(0.113)	(0.070)	(0.789)	(0.826)	(0.574)	(0.778)	**(0.001)**
*V*. *cholerae* (cor. coefficient)	0.974	-0.166	0.430	-0.301	0.694	0.999*	-0.165	-0.176	0.233	0.012	0.958*
*V*. *cholerae* (p value)	(0.145)	(0.754)	(0.395)	(0.562)	(0.126)	**(0.033)**	(0.755)	(0.739)	(0.658)	(0.981)	**(0.003)**
*V*. *vulnificus* (cor. coefficient)	0.974	0.028	0.509	-0.109	0.459	0.999*	-0.091	-0.278	-0.063	0.624	0.653
*V*. *vulnificus* (p value)	(0.145)	(0.958)	(0.303)	(0.838)	(0.360)	**(0.033)**	(0.864)	(0.593)	(0.905)	(0.186)	(0.160)
*P*. *aeruginosa* (cor. coefficient)	0.977	0.240	0.312	0.181	-0.081	0.999*	0.048	-0.241	-0.377	0.991*	-0.079
*P*. *aeruginosa* (p value)	(0.137)	(0.646)	(0.547)	(0.731)	(0.879)	**(0.025)**	(0.928)	(0.646)	(0.462)	**(0.000)**	(0.881)
*Salmonella spp*. (cor. coefficient)	0.974	-0.218	0.359	-0.339	0.708	0.999*	-0.175	-0.122	0.314	-0.205	0.970*
*Salmonella spp*. (p value)	(0.145)	(0.678)	(0.485)	(0.511)	(0.116)	**(0.033)**	(0.741)	(0.818)	(0.545)	(0.697)	**(0.001**)

P-values are represented in parenthesis with

*P<0.05.

**Fig 6:**
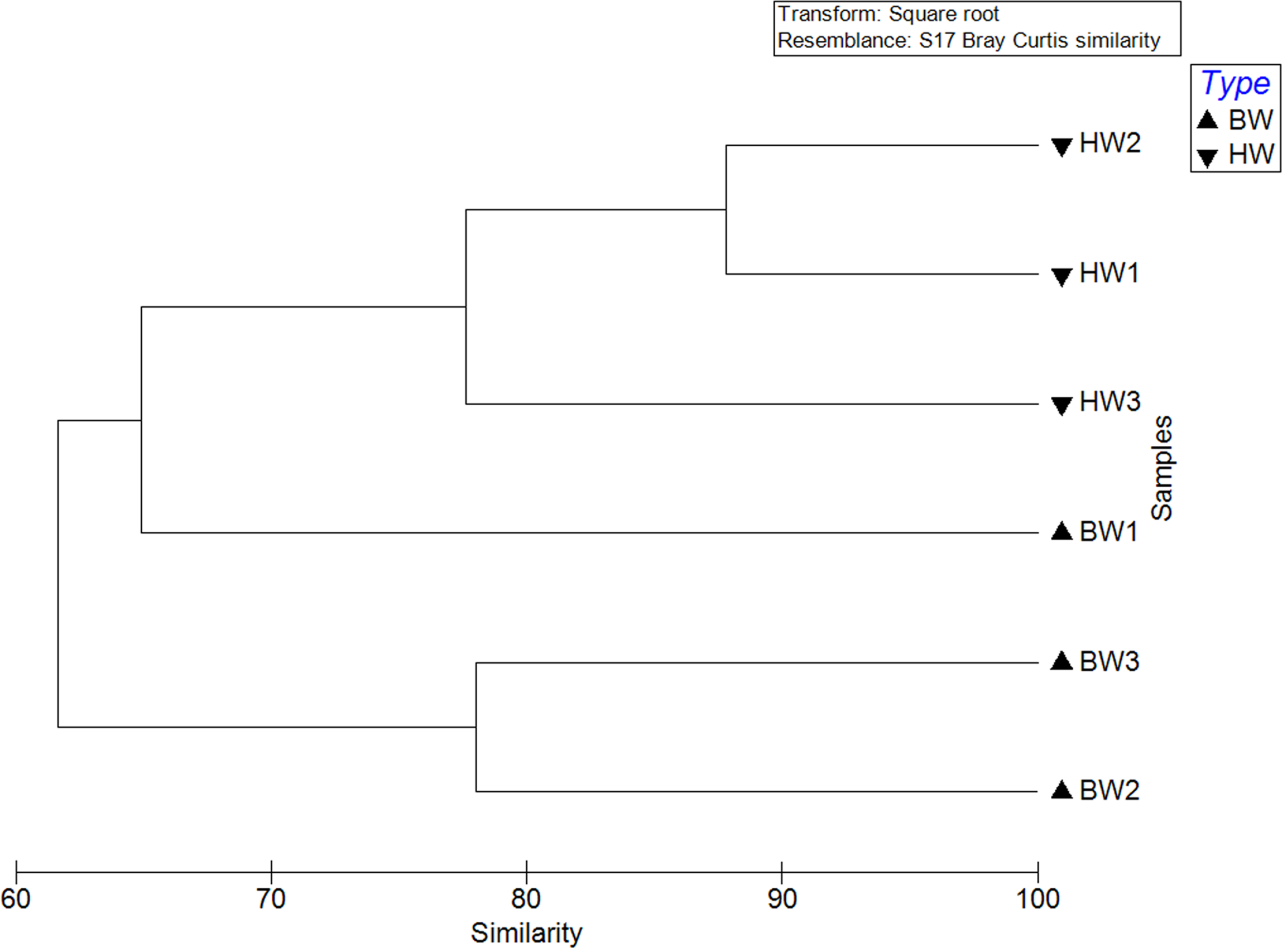
Clustering analysis of microbial composition in ballast and harbor waters based on Bray-Curtis similarity index.

A clustering analysis showed that the microbial community in the harbor waters was more similar in microbial composition than ballast waters at a similarity cut off of 75% (Fig 6). At the same level of similarity, BW2 and BW3 showed more resemblance compared to BW1, and this clustering pattern observed on a Principal Component Analysis (PCA) plot was statistically verified using an ANOSIM analysis (P<0.05).

**Fig 7:**
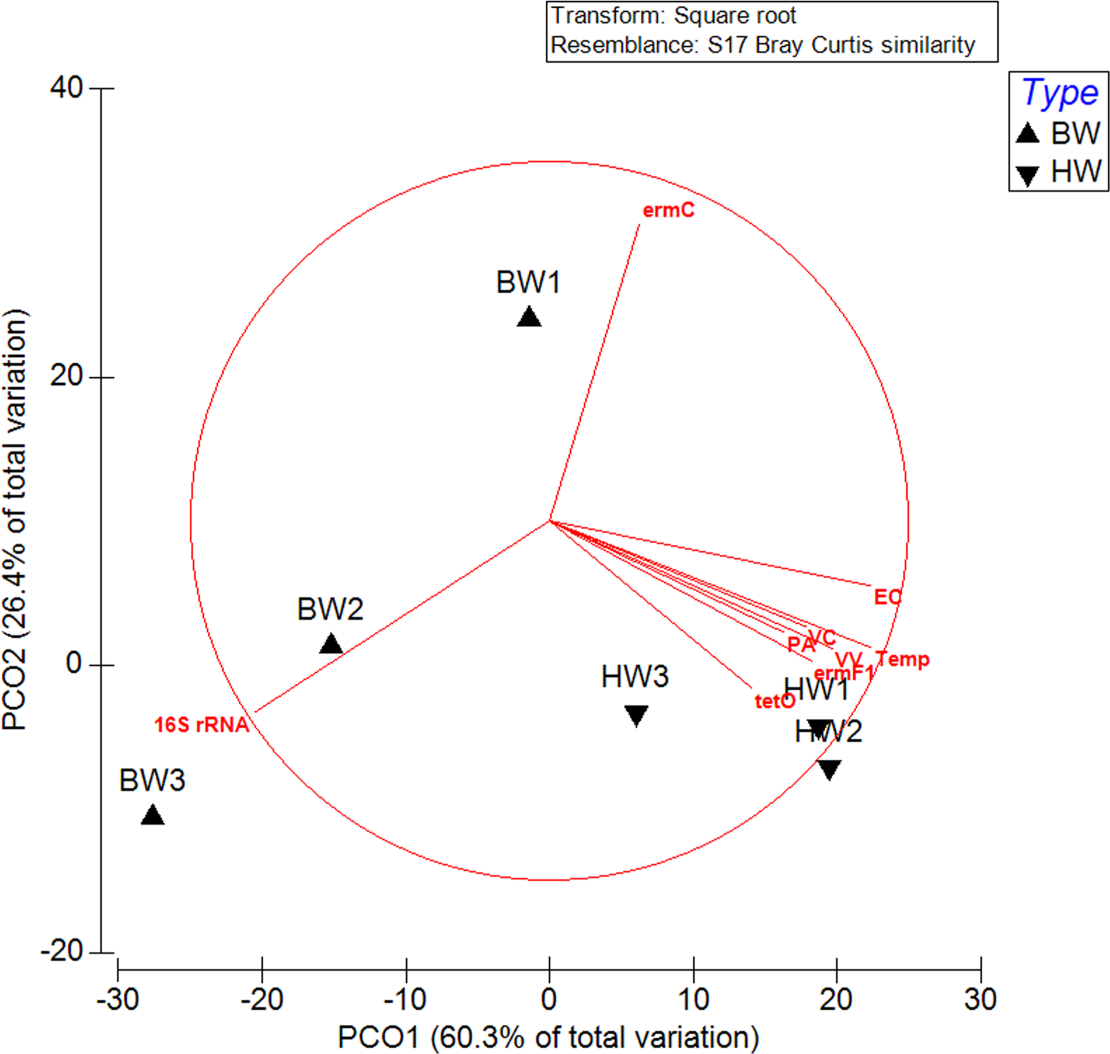
Principal component analysis (PCA) of the microbial community structure of ballast and harbor waters in relationship with pathogen, ARB and ARG data. Only variables with a Pearson correlation coefficient of >0.7 were included in the PCA plot. EC–*E*. *coli*, VC–*V*. *cholera*, *P*. *aeruginosa*, VV–*V*. *vulnificus*, Temp–temperature.

Using the PERMANOVA analysis in the Primer v6 software, microbial community patterns in the harbor samples were positively correlated (using a multivariate analysis where Pearson correlation coefficient of >0.7) to measurements of *E*. *coli*, *V*. *cholera*, *P*. *aeruginosa*, *V*. *vulnificus*, temperature, *tetO* and *ermF1* genes (Fig 7). The PCO1 and PCO2 explained 60.3% and 26.4% of the variation. The ARG *ermC* was positively correlated to microbial composition of BW1, while BW2 and 3 correlated to 16S rRNA gene sequence (Fig 7).

## Discussion

Mid ocean exchange (MOE) of ballast waters has been implemented by IMO to prevent the dissemination of invasive aquatic species. This method requires ships to exchange ballast water at least 200 nautical miles away from shore at an ocean depth of at least 200 m. It is proposed that replacement of ballast water with mid ocean waters (containing oligotrophic marine bacteria) flushes or dilutes any potential coastal dwelling invasive organisms, which may have been picked up during the voyage of ships. In a few other studies, this method of water replacement has shown no significant decrease in bacterial counts (pre- versus post-exchange). Rather, a decline in bacterial abundance in ballast waters was mainly attributed to longer water retention time in ballast tanks [8, 35, 36]. Overall, two of the ballast waters in our study had a significant lower abundance of viable HPC than harbor waters (with exceptions of BW2 and HW2). In an earlier study using flow cytometry, Joachimsthal et al. (2003) reported bacterial concentrations of ballast waters in Singapore ranging from 2.35 × 10^6–^5.87 × 10^7^ cells/mL, with differences between harbor and ballast waters attributed to differences in location and season of ballast water uptake and open ocean water samplings [37, 38].

In our study, the levels of indicator organisms measured in ballast waters were within limits administered by IMO, with the exception of one ballast water sample where *Enterococcus* concentrations exceeded guideline values. In comparison to another study using FISH techniques, levels of *Vibrio spp*. and *E*. *coli* in our study was generally much lower than previously reported [12]. We found a correlation between higher temperatures, turbidity and *V*. *vulnificus* abundance in harbor waters (P<0.05). Studies have shown that marine pathogens such as *Vibrio spp*. may preferentially thrive in coastal areas with increased water temperatures and reduced salinity (NaCl <25 ppb, [39]) due to terrestrial runoff.

*Pseudomonas spp*. and *Salmonella spp*. are not listed as ballast water indicator organisms of concern according to IMO guidelines. However, we selected these bacteria for testing based on detection in other ballast water studies. For example, Burkholder et al. (2007) reported the presence of other pathogens including *P*. *aeruginosa* in 48% of ballast waters surveyed from vessels in the east and west coast of the United States [40]. In another study using chromogenic agar, Altug et al. (2012) were able to detect viable *Salmonella spp*. (300^–^500 CFU/100 mL) in ballast waters coming from different regions in the world but not in the Sea of Marmara, Turkey where the ships docked [41]. For this study, although *Pseudomonas spp*. in ballast waters were detected at levels lower (0^–^20 GC/mL) relative to the other indicator organisms, their ability to form biofilms on the surfaces of tanks and resistance to environmental stress make them important targets to monitor [42].

Compared to soil, antibiotic resistance of bacteria in marine environments has not been well documented. The ocean is a natural habitat of antibiotic-producing bacteria with marine aquaculture introducing more antibiotics to treat infections and boost aquacuture production. Plasmids are mobile vectors for the transfer of resistance genes that confer an advantage to their host’s adaptation to different ecological niches [43]. In this study, samples were analyzed for the presence of ARB and associated ARGs, which are increasingly being recognized as xenobiotics, and emerging contaminants in the aquaculture industry. As a whole, the culturable marine microbial communities in ballast and harbor waters were most resistant to trimethoprim, sulfanilamide, lincomycin and tetracycline. Shah et al. (2014) reported the same patterns of ARB (by proxy of pheno- and genotypic screening) in Chilean marine sediments of salmon fish farms, which suggests that aquaculture activity likely contributes to antibiotic resistance patterns in the marine environment [44]. Interestingly, BW1 had the lowest levels of phenotypic resistance to all antibiotics tested, which could be attributed to its more complex and diverse microbial community structure resulting in its distinct characteristics compared to the other samples. The ARB population in BW1 samples could be more fastidious in nature, incapable of growing on marine media supplemented by antibiotics. This reasoning is supported by comparing the pheno- and genotypic data which shows the identification of a wide array of ARG targets in BW1 compared to the other samples. This highlights the importance of using both culturing and screening of antibiotic resistance gene markers for a more thorough assessment of antibiotic resistance given that pheno- and genotypic results may contradict each other due to confounding effects of cell viability and other growth impediments.

In most of the samples for which ARGs were detected, higher concentrations were found in the harbor water samples than a non-impacted freshwater body in Singapore, where ARG concentrations were at least half or those reported in the harbor samples (non-impacted site: *sul1*–1.11 x 10^2^ gene copies/mL, *cfr*–below detection limit). The persistence of *sul1* and *dfrA* genes together with the relatively high levels of viable ARB counts (for most but not all of the samples), suggests successive pressure exerted by sulfamethoxazole, trimethoprim or co-trimoxazole, a combination of the former two antibiotics. Sulfonamide and tetracycline are commonly used to treat bacterial and protozoan infections in aquaculture leading to the prevalent problem of resistant bacteria in these environments [45–47]. Even without selective pressure, sulfonamide-resistant bacteria can remain stable in the aqueous environment for 5 to 10 years [48].

Other genes including *tetM*, *crf*, *ermB1*, *C*, *F1* and *G* were also identified at lower concentrations in all samples except for BW3 and HW3. Most marine environmental surveys, particularly in fish farms, have identified tetracycline, chloramphenicol and macrolide resistant gene markers [45, 49–52]. Strong positive correlations were shown between *cfr* and *P*. *aeruginosa* gene copies (in HW2), and *E*. *coli* and *ereA* gene copies (in HW1) while ARGs *ermG* and *tetO* correlated with a combination of at least four of the indicator organisms tested in this study. These correlation patterns suggest that these indicator organisms may be contributing to the resistome of coastal environments. Plasmids carrying the *cfr* gene mediating resistance to linezolid and chloramphenical have only been decribed in *Staphylococcus* and *Enterococcus* [53] but not in other taxa of pathogens. Likewise, there has been no reported association of resistance to macrolides in *Vibrio spp*. encoded by the *ermG* gene. In aquacuture sources, it has been observed that genera of Pseudomonas carry florfenicol resistant genes [49]. It would be worthwhile to taxonomically identify a variety of antibiotic resistant phenotypes and test for the presence of these ARGs to verify this correlation. Determining this would enable us to estimate the risk of dissemination of ARGs between the environmental reservoir and human pathogens.

The PCA analysis showed a tighter clustering of the microbial composition of harbor waters indicating higher similarities in structure as compared to the ballast waters which are more dispersed. Although there are some distinguishing bacterial populations among the ballast water samples, it is apparent that diversity is largely dependent on the site of water uptake. The harbor waters were more similar in composition and showed significant positive correlations to indicator organisms (*E*. *coli*), and pathogens (*V*. *cholera*, *P*. *aeruginosa*, *V*. *vulnificus*), temperature and ARGs (*tetO* and *ermF1* genes).

By comparing microbial communities of ballast and harbor waters, we found low levels of similarity at the species level, which is expected given that the harbor samples are more terrestrially impacted than off-shore oligotrophic seawater samples. At the phyla level, Proteobacteria dominated across all samples, with SAR11 showing notably higher levels of abundance in harbor waters. Members of the SAR11 clade are ubiquitous, and highly adapted to nutrient poor oligotrophic environments such as surface waters of open oceans [54]. Open ocean SAR11 populations, unlike their coastal counterparts, exhibit diel oscillations at the transcript level and may regulate light-dark cycles of transcriptional activity by tracking surrounding environmental conditions [55]. Steindler et al. (2011) found that in light-deprived, nutrient starved conditions, *Candidatus Pelgibacter ubique*, a member of the SAR11 clade, are able to utilize carbon reserves, shrinking to a volume of ~0.014 μm^3^ [56]. The marine cyanobacteria, Synechococcus, were also detected at higher levels in harbor waters. The dominance of phototrophs in the harbor waters versus ballast waters (where illumination is absent in the tanks) is influenced by sampling in the late morning at the surface, within the photic zone of the water column. In contrast, nutrient and light-stress in ballast tanks have been shown to take a toll on autotrophic nanoflagellates and mixotrophic populations, with the loss of photosynthetic pigments and dramatic decreases in counts over time [35]. Together, these findings offer a rational explanation for observed low levels of phototrophic cyanobacteria in the ballast water samples and also may suggest a selection of open ocean SAR11 phenotypes.

Taxa associated with marine hydrocarbon degradation (Oceanspirillales, Thiotrichales, Flavobacteria) were enriched in the ballast water samples. Members of these taxa are capable of hydrocarbon oxidation, and have been identified in sediments of marine hydrocarbon seeps from 16S rRNA sequence clone libraries of environmental samples [57]. BW1 had the highest microbial diversity with high abundances of Caldilineales and Sphingobacteriales, which are commonly found in activated sludge in wastewater treatment plants [58, 59]. Over summer periods, Zhang et al. (2014) detected the prevalence of Caldilineales in the subtropical Pearl River Estuary sediments which receives large volumes of inflow from the South China Sea and anthropogenic inputs from surrounding industries [60]. Interestingly, the last point of ballast water uptake for BW1 was in the South China Sea which possibly suggests that a combination of an upwelling event, sediment dispersal and water circulating patterns facilitated the hitch-hiking of these microbes in ballast water from this point of origin to our local ports.

The habor waters showed more similarity in microbial community structure while the ballast waters had more variability. This is expected given that ballast water of the ships surveyed were situated at diferent locations during uptake. Incorporating methods of assessing microbial community composition by next-generation sequencing (NGS) techniques, in tandem with monitoring indictor organisms and nutrient inputs with the effect on microbial growth and potential harmful algal provides a comprehensive approach of understanding ecological impact of shipping activity and ballast water dispersal.

In conclusion, we observed differences in the bacterial community composition, indicator organisms (*E*. *coli*, *Enterococcus*, *P*. *aeruginosa*, *Salmonella spp*., *Vibrio spp*.), and the concentrations of ARB and ARGs in ballast water of 3 ships and harbor waters at their port of call. Harbor waters exhibited a general trend of higher concentrations of indicator organisms, however we detected the presence of *Enterococcus* in all ballast water samples, with one sample, in particular, exceeding levels recommended by IMO. We found correlations between the concentrations of indicator organisms (*E*. *coli*, *V*. *cholera*, *V*. *vulnificus*, *P*. *aeruginosa*) and ARGs, which suggests that these species in ballast and harbor waters may be carriers of ARGs. Selective culturing, isolation and antibiotic susceptibilty testing of these marine species will be required to confirm if these potential pathogens are indeed resistant and carriers of ARGs. We applied NGS, different molecular assays and conventional microbiological methods to provide detailed microbial measurements and other predictive parameters which may dictate the threat to microbiological safety standards in ballast waters.

## Supporting Information

S1 Fig Rarefaction curves of species richness (97% cut off) in ballast and harbor waters. (TIF)
