# Correction: A Comparison of Microbial Water Quality and Diversity for Ballast and Tropical Harbor Waters

**DOI:** 10.1371/journal.pone.0154652

**Published:** 2016-04-26

**Authors:** Charmaine Ng, Thai-Hoang Le, Shin Giek Goh, Liang Liang, Yiseul Kim, Joan B. Rose, Karina Gin Yew-Hoong

The following information is missing from the Funding section: This work was supported in collaboration with the National Science Foundation, Partnerships for International Research and Education (OISE-0530174).

There is an error in the first sentence of the third paragraph in the Introduction. The correct sentence is: In a study of bacteriological assessment of ballast waters of six ships docked at Singapore harbors, Joachimsthal et al. (2004) used fluorescence in situ hybridization (FISH) coupled with flow cytometry to enumerate total bacteria, and fluorescent tags to differentiate between Enterobacteria, *Vibrio spp*., and *E*. *coli*.

There is an error in the caption for Fig 5, “The abundance of the ARGs in ballast and harbor waters. (A) BW and HW1, (B) BW2 and HW2, (C) BW3 and HW3.”

**Fig 5:**
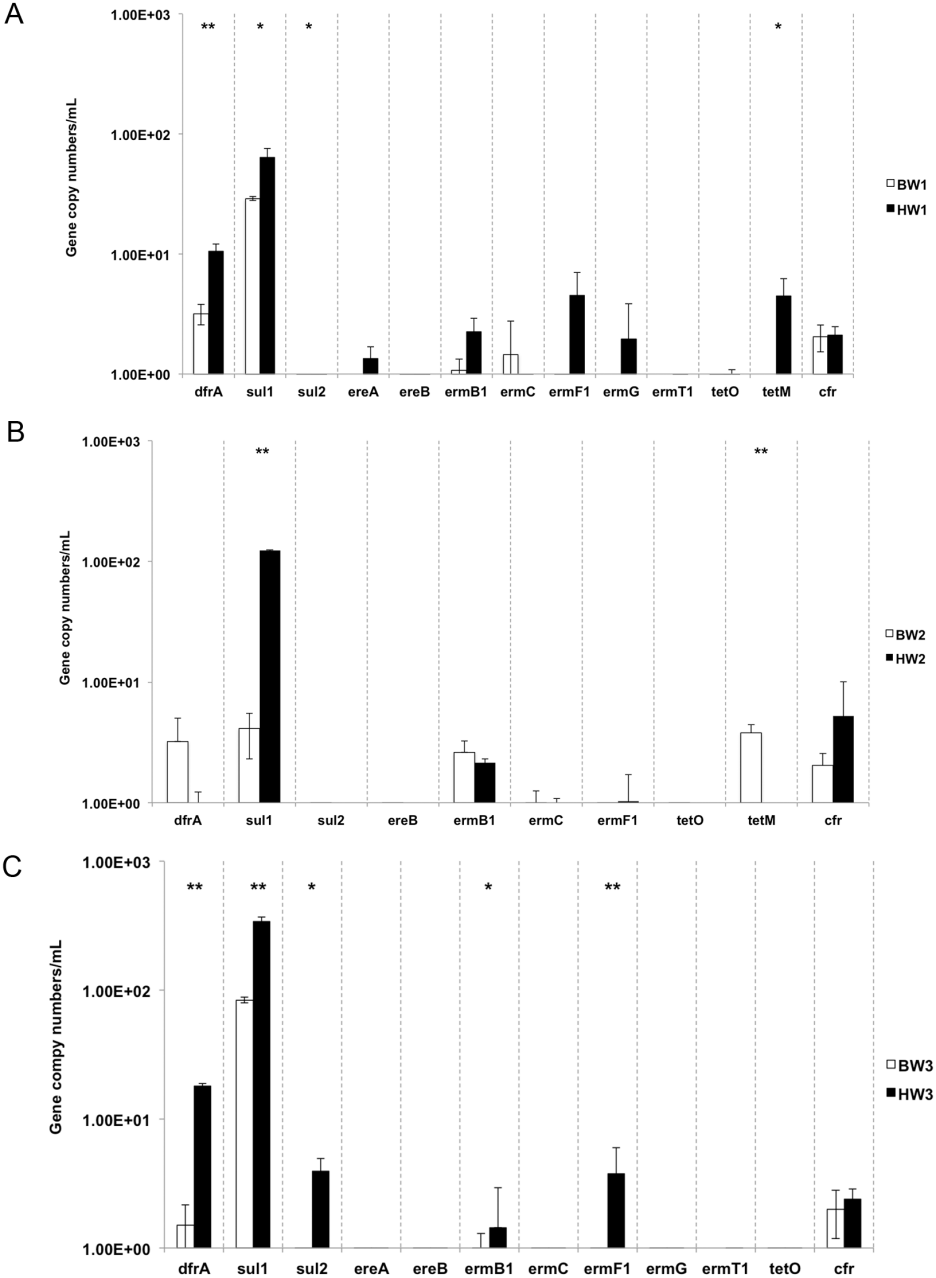
The abundance of the ARGs in ballast and harbor waters. (A) BW1 and HW1, (B) BW2 and HW2, (C) BW3 and HW3. Two-tailed T-test, *P<0.05, **P<0.01. ARGs with non-detects in both BW and HW samples were excluded.

Please see the complete, correct Fig 5 caption here.

## References

[1] Ng C, Le T-H, Goh SG, Liang L, Kim Y, Rose JB, et al (2015) A Comparison of Microbial Water Quality and Diversity for Ballast and Tropical Harbor Waters. PLoS ONE 10(11): e0143123 doi: 10.1371/journal.pone.0143123 26575481 10.1371/journal.pone.0143123 PMC4648578

