# Supplementary figures and images for: A Comparison of Microbial Water Quality and Diversity for Ballast and Tropical Harbor Waters
Source: PLoS One. 2015 Nov 17;10(11):e0143123. doi: 10.1371/journal.pone.0143123 (PMC4648578; doi:10.1371/journal.pone.0143123)

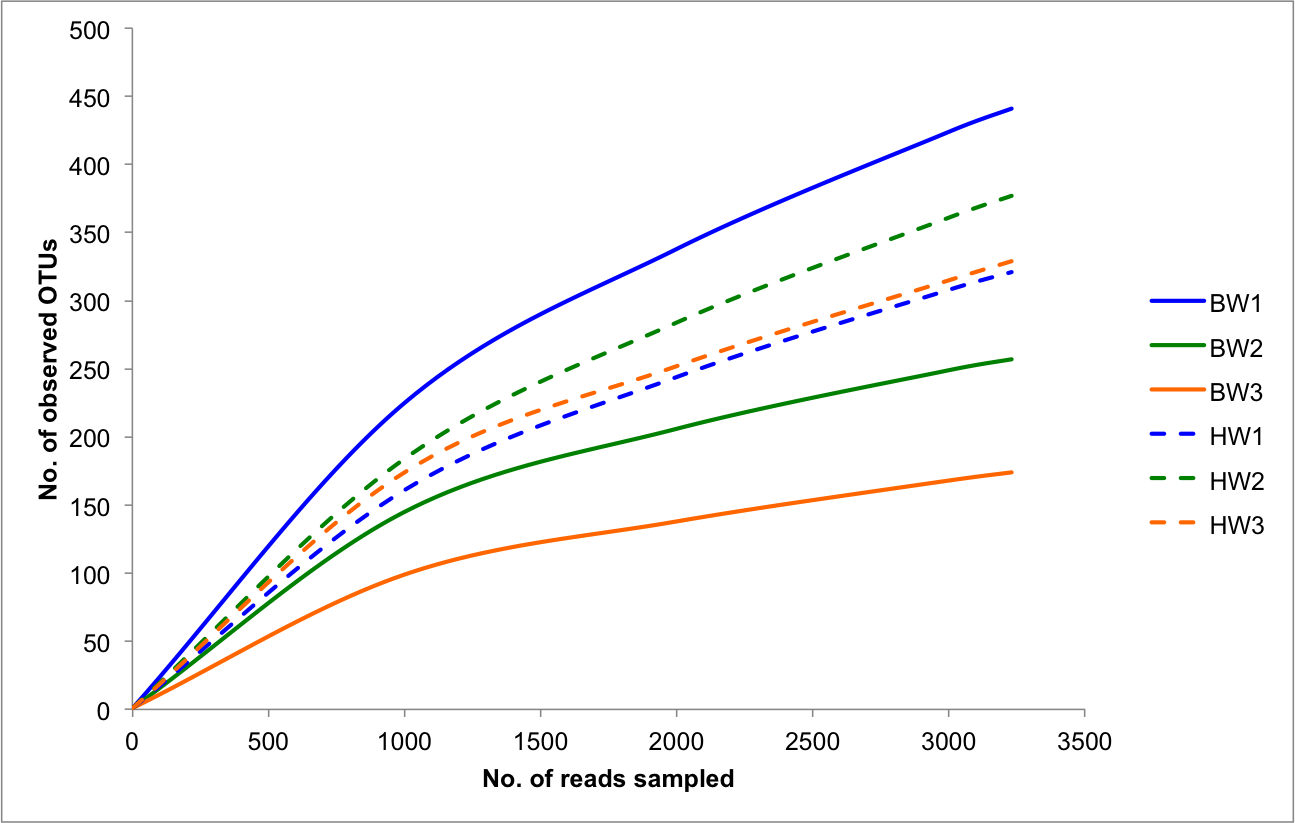

Supplement: S1 Fig — (TIF) [file pone.0143123.s001.tif]
